# Temporal reproducibility of IgG and IgM autoantibodies in serum from healthy women

**DOI:** 10.1038/s41598-022-10174-3

**Published:** 2022-04-13

**Authors:** T. V. Clendenen, S. Hu, Y. Afanasyeva, M. Askenazi, K. L. Koenig, T. Hulett, M. Liu, S. Liu, F. Wu, A. Zeleniuch-Jacquotte, Y. Chen

**Affiliations:** 1 Department of Population Health, Division of Epidemiology, NYU Langone Health School of Medicine, 180 Madison Ave. 5th Floor, New York, NY 10016 USA; 2 CDI Laboratories, Baltimore, MD USA

**Keywords:** Proteomic analysis, Biomarkers, Risk factors

## Abstract

Autoantibodies are present in healthy individuals and altered in chronic diseases. We used repeated samples collected from participants in the NYU Women’s Health Study to assess autoantibody reproducibility and repertoire stability over a one-year period using the HuProt array. We included two samples collected one year apart from each of 46 healthy women (92 samples). We also included eight blinded replicate samples to assess laboratory reproducibility. A total of 21,211 IgG and IgM autoantibodies were interrogated. Of those, 86% of IgG (*n* = 18,303) and 34% of IgM (*n* = 7,242) autoantibodies showed adequate lab reproducibility (coefficient of variation [CV] < 20%). Intraclass correlation coefficients (ICCs) were estimated to assess temporal reproducibility. A high proportion of both IgG and IgM autoantibodies with CV < 20% (76% and 98%, respectively) showed excellent temporal reproducibility (ICC > 0.8). Temporal reproducibility was lower after using quantile normalization suggesting that batch variability was not an important source of error, and that normalization removed some informative biological information. To our knowledge this study is the largest in terms of sample size and autoantibody numbers to assess autoantibody reproducibility in healthy women. The results suggest that for many autoantibodies a single measurement may be used to rank individuals in studies of autoantibodies as etiologic markers of disease.

## Introduction

Novel technologies have led to the recognition that numerous antibodies against self molecules, autoantibodies (AAbs), are present in healthy, free of auto-immune diseases, individuals^1,2^ AAbs are highly individualized, accumulate throughout life and have been observed to increase with age, including among individuals without autoimmune disease^3^. Some AAbs, particularly IgMs, are characterized by poly-affinity for various antigens, though many highly specific and high binding affinity IgG and IgM AAbs have been identified^4^. AAbs are thought to play a role in apoptosis and apoptotic waste cleanup and may have a role in the regulation of some anti-inflammatory responses^4–6^.

It has been hypothesized that AAb profiles may be altered in various diseases, including cardiovascular disease, dementia, and cancer^7–9^. AAbs are thought to play a role in the recognition and removal of malignant and pre-cancerous cells^9^. To be able to interpret the presence and patterns of AAbs in patients with various diseases, it is important to identify AAbs that are commonly found in healthy individuals. Estimates of the stability of AAbs over time in healthy individuals are also needed to conduct prospective studies focusing on incidence of chronic diseases. AAbs that are stable in healthy individuals but increase or decrease when individuals develop a specific disease may be good candidate biomarkers for early detection or progression of disease.

Only with the recent development of auto-antigen arrays, has it been possible to examine large numbers of AAbs in healthy individuals. Nagele et al.^3^ observed an abundance of AAbs (IgG) in 57 healthy individuals on a 10,000 human protein microarray. Two studies from the same group examined IgG autoreactivity to 335 protein fragments and found that AAb profiles were relatively stable over one year in samples collected from four individuals^3,10^. To date, there are no studies that include large numbers of IgG AAbs and longitudinal samples, and no characterization of IgM AAb profiles in healthy women.

To address this knowledge gap, we conducted a study to identify both IgM and IgG AAbs commonly found in healthy women, using arrays of over 21,000 proteins and protein fragments (herein referred to as autoantigens), and examined the temporal reproducibility of their measurement using longitudinally collected samples.

## Methods

### Study subjects

We used blood samples from participants in the NYU Women’s Health Study. The NYU Women’s Health Study is a cohort of 14,274 women enrolled between 1985 and 1991 at a breast cancer screening center in New York City. Participants are followed up through self-completed questionnaires every few years and linkage with the National Death Index and tumor registries of New York, New Jersey, and Florida (where over 85% of the participants reside). We collected blood samples at enrollment and at annual repeat screening visits until 1991. Serum samples were stored at −80 °C. Two or more yearly serum samples are available for 52% of the participants. We selected 46 participants in the NYUWHS who: (1) donated two or more blood samples at 1-year intervals; (2) had not been diagnosed with cancer or CVD up to last complete follow-up (2016). This study was approved by the NYU Langone School of Medicine Institutional Review Board in accordance with the policies and regulations governing research with human subjects. Informed consent was obtained from all participants in the NYUWHS.

### Assays

Assays were conducted using HuProt arrays (version 3.1) provided by CDI Laboratories, Inc^11^. Each array comprised 22,817 human proteins, isoform variants, or protein fragments (thereafter referred to as autoantigens). Sera were diluted 1:1,000 and reacted with individual HuProt protein microarrays. Briefly, the HuProt arrays were blocked with blocking solution (5% BSA/1 × TBS-T) at room temperature for 1 h, and then probed with serum samples (diluted 1:1000) at 4 °C overnight. The arrays were then washed with 1 × TBS-T for 3 times, 10 min each, and probed with Alexa-647 labeled anti-human IgG Fc gamma fragment specific and Cy3 labeled anti-Human IgM Fc5μ fragment specific secondary antibodies (Jackson ImmunoResearch, West Grove, PA) at room temperature for 1 h, followed by three washes of 1 × TBS-T, 10 min each, and then dried and scanned using a GenePix 4000B scanner (Molecular Devices, Sunnyvale, CA) to create raw TIF images. Spots were then aligned to HuProt array list (.gal) files, and spot readings captured as raw Genepix results (.gpr) files used for data processing. In total, 21,211 autoantigen spots (93%) passed laboratory quality control criteria for spot quality and had their identities confirmed by the laboratory. We focus on that subset for this study.

Eight quality control replicates from a common pool generated using samples from eight healthy NYU Women’s Health Study participants were included to assess assay variability. Samples were randomly ordered for laboratory analyses. Samples were identified only by a sample number, so that laboratory personnel could not identify which samples were from the same woman nor which were QC samples.

### Data processing

For each probe, the signal intensity is given by the median foreground by Genepix. Because each protein was printed in duplicate, the mean of the two readings was used. Signal intensity values were log-transformed (base two).

### Statistical analysis

Laboratory variability was assessed by means of the coefficient of variation (CV) using data from the eight blinded replicate quality control samples. All subsequent analyses were limited to AAbs with CV < 20%. The intraclass correlation coefficient (ICC) was used to examine temporal reproducibility of the log2-transformed signal intensity for all AAbs. The ICC estimates the fraction of the total variation (within-subject plus between-subject) that is due to between-subject variation^12,13^. Heatmaps were created using log2-transformed non-normalized data to examine clustering of samples across AAbs using the R package “ComplexHeatmap” and Pearson distance hierarchical clustering of samples and AAbs. We also conducted analyses using quantile normalization in the R package “normalize.quantiles” on the log2-transformed data because this method is commonly used to reduce assay variability^18^. To assess the correlation between AAb measurements and the covariates age and BMI, we calculated Pearson correlation coefficients of the average value of the two log2-transformed repeat AAb measurements with the average log-transformed age and BMI from the two visits.

### Data availability

The data generated in this study are available within the article and its supplementary data files.

## Results

The 46 women included in this study were between the ages of 38 and 68 (mean age 51.7), and 50% were premenopausal. BMI ranged from 19.2 to 35.4 kg/m^2^ (mean BMI 24.1). Twenty-nine (63%) women were never smokers, 10 (22%) were former smokers, and 7 (15%) were current smokers at both blood donations. Consistent with the distributions observed in the full NYUWHS cohort, many of the women included in this analysis reported their race/ethnicity as White (70%, *n* = 32 women), 15% Black (*n* = 7), 4% Asian, and 4% Hispanic (6% of women did not report).

Table 1 reports CVs estimated using the eight blinded replicates from a common sample pool, randomly interspersed among the study samples. A high percentage of the IgG AAbs (86%) showed acceptable lab reproducibility (CV < 20%) and 25% showed high lab reproducibility (CV < 10%). However, less than half (34%) of the IgM AAbs had a CV < 20% and only 0.4% a CV < 10%.

**Table 1:** Coefficients of variation, *n* = 8 replicate QC samples (raw data).

	Total *N*	CV, *n* (%)			
		< 10%	10–15%	15–20%	≥ 20%
IgG	21,200	5344 (25.2)	8560 (40.4)	4399 (20.8)	2897 (13.7)
IgM	21,209	83 (0.4)	1762 (8.3)	5397 (25.4)	13,967 (65.9)

11 IgG autoantigen probes and 2 IgM had no variation and were not included in the above table.

ICCs were estimated, and heatmaps generated, for the 18,303 IgG and 7242 IgM AAb with acceptable lab reproducibility (CV < 20%). A very high proportion of both of IgG and IgM AAbs (76% of IgG and 98% of IgM) showed excellent temporal reproducibility (ICCs > 0.8, Table 2). ICCs computed using quantile-normalized data were lower than the ICCs computed from the raw data (log2-transformed intensities) (Table 2). However, a high proportion of IgG (69%) and a substantial proportion of IgM (40%) AAbs still had an ICC ≥ 0.6 (Table 2).

**Table 2:** Intra-class correlation coefficients (limited to autoantibodies with CV < 20%).

	*N*		ICC, *n* (%)					
			≤ 0.59		0.6– < 0.79		0.8–1.0	
IgG	18,303							
		Log_2_ scale	871	(4.76)	3465	(18.9)	13,967	(76.3)
		Quantile normalized	5736	(31.3)	5460	(29.8)	7107	(38.8)
IgM	7242							
		Log_2_ scale	14	(0.19)	131	(1.81)	7097	(98.0)
		Quantile normalized	4395	(60.7)	1861	(25.7)	986	(13.6)

**Figure 1:**
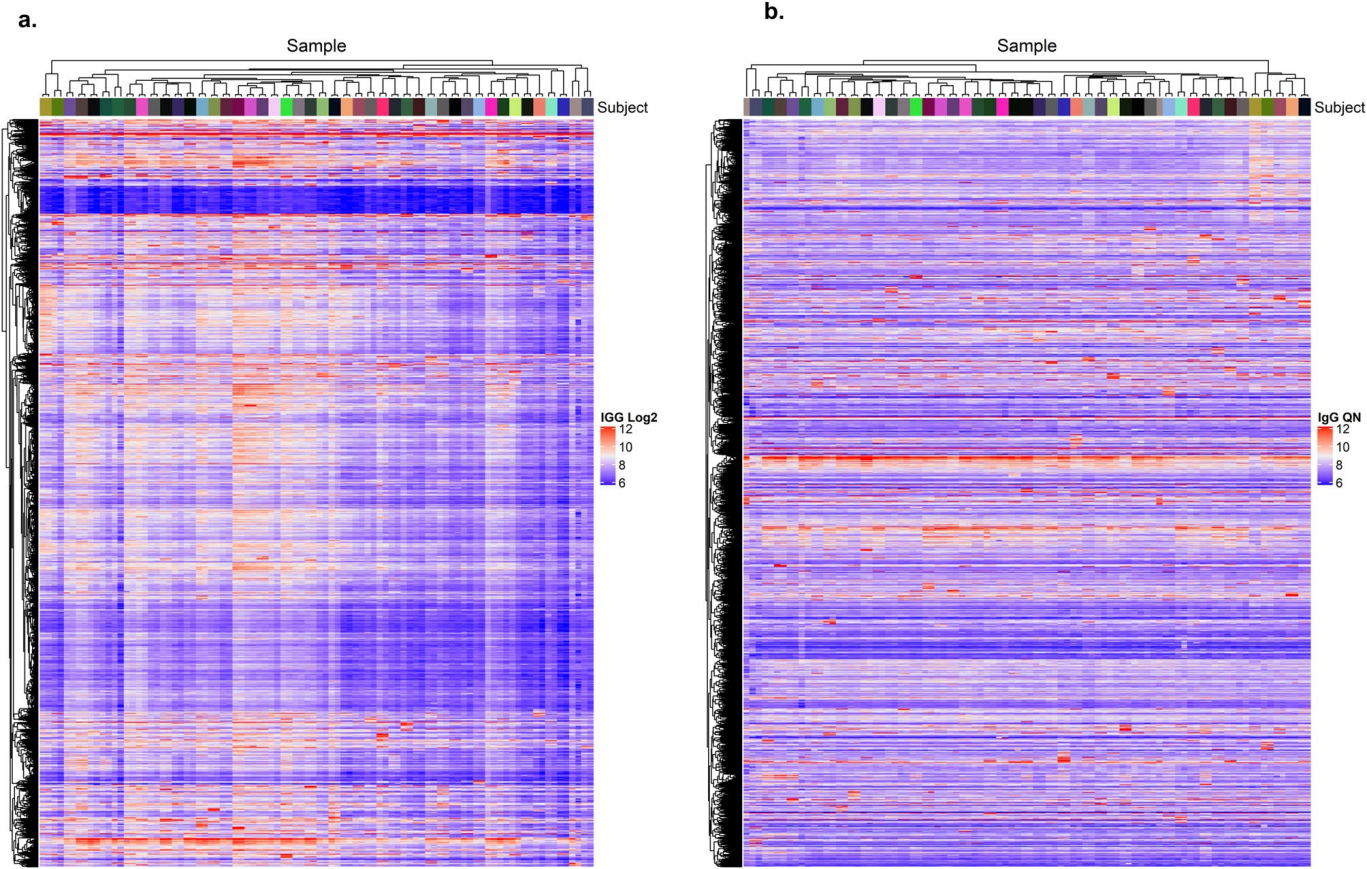
Heatmap of IgG autoantibodies, log2 transformed (left) and quantile normalized (right) (*n* = 18,303 with CV < 20%). IgG autoantibody profiling using HuProt arrays of repeat samples collected one year apart from *n* = 46 heathy subjects (*n* = 92 samples total). Raw log2 transformed data (**a**) and quantile normalized data (right) was used to cluster AAbs and samples using pearson distance higherarchical clustering for 18,303 probes with CV < 20%. Top bar annotation colors correspond to individual subjects. (Two samples from each subject clustered together for all 46 subjects using log2 transformed data and for all but one subject using quantile normalized data).

**Figure 2:**
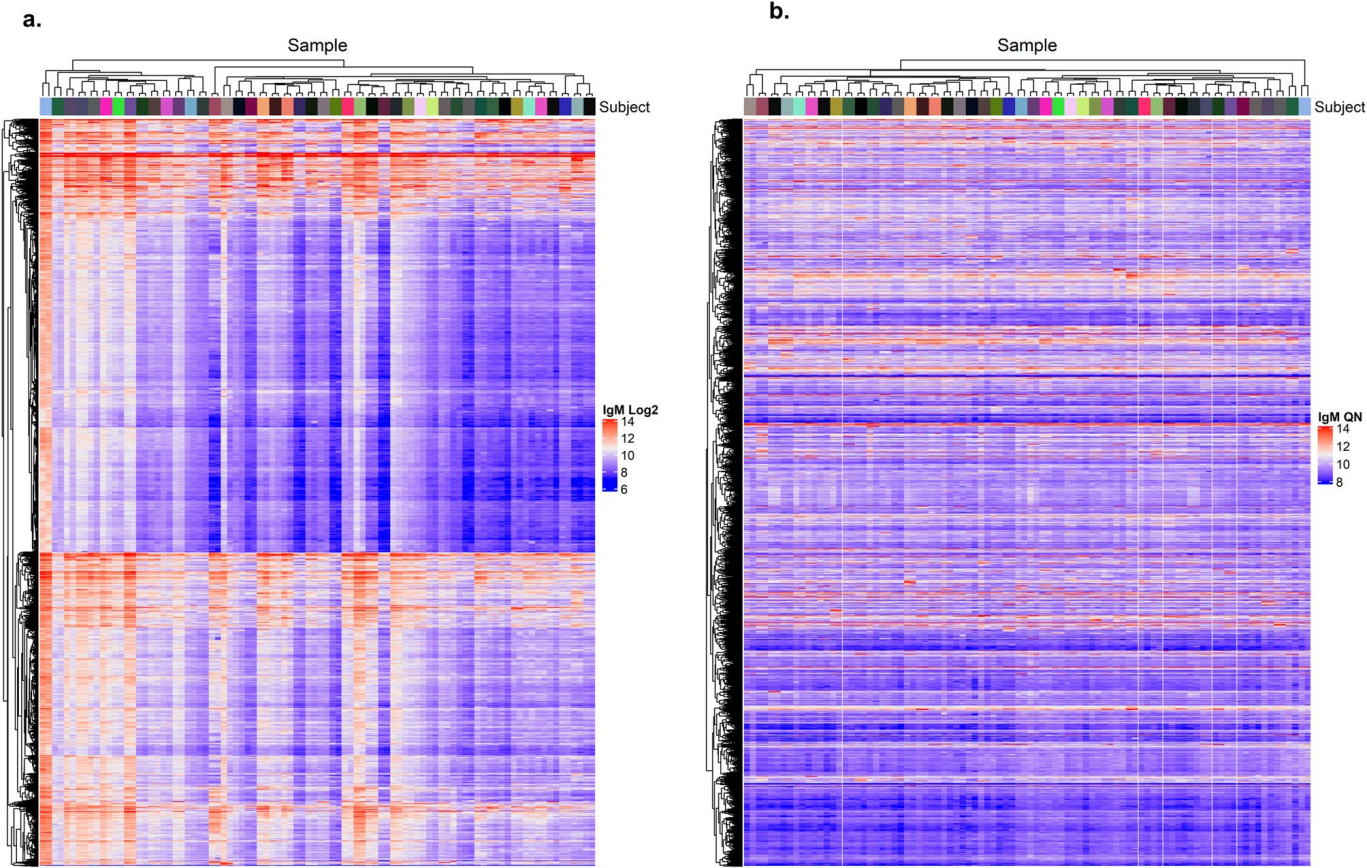
Heatmap of IgM autoantibodies, log2 transformed (left) and quantile normalized (right) (*n* = 7,242 with CV < 20%) IgM autoantibody profiling using HuProt arrays of repeat samples collected one year apart from *n* = 46 heathy subjects (*n* = 92 samples total). Raw log2 transformed data (**a**) and quantile normalized data (right) was used to cluster AAbs and samples using pearson distance higherarchical clustering for 7,242 probes with CV < 20%. Top bar annotation colors correspond to individual subjects. (Two samples from each subject clustered together for all 46 subjects using both log2 transformed data and quantile normalized data).

Heatmaps showing sample clustering (columns) and probe clustering (rows) based on log2-transformed signal intensity (left) and quantile normalized log2-transformed signal intensity (right) for AAbs with CV < 20% are shown in Fig. 1 for IgG and Fig. 2 for IgM. The heatmaps show that IgG and IgM AAb profiles were much more similar for samples collected from the same woman one year apart than for samples collected from different women. This was true both before and after quantile normalization, though clustering was perfect before normalization using both IgG and IgM while one set of paired samples did not cluster together after quantile normalization in the IgG analysis.

**Figure 3:**
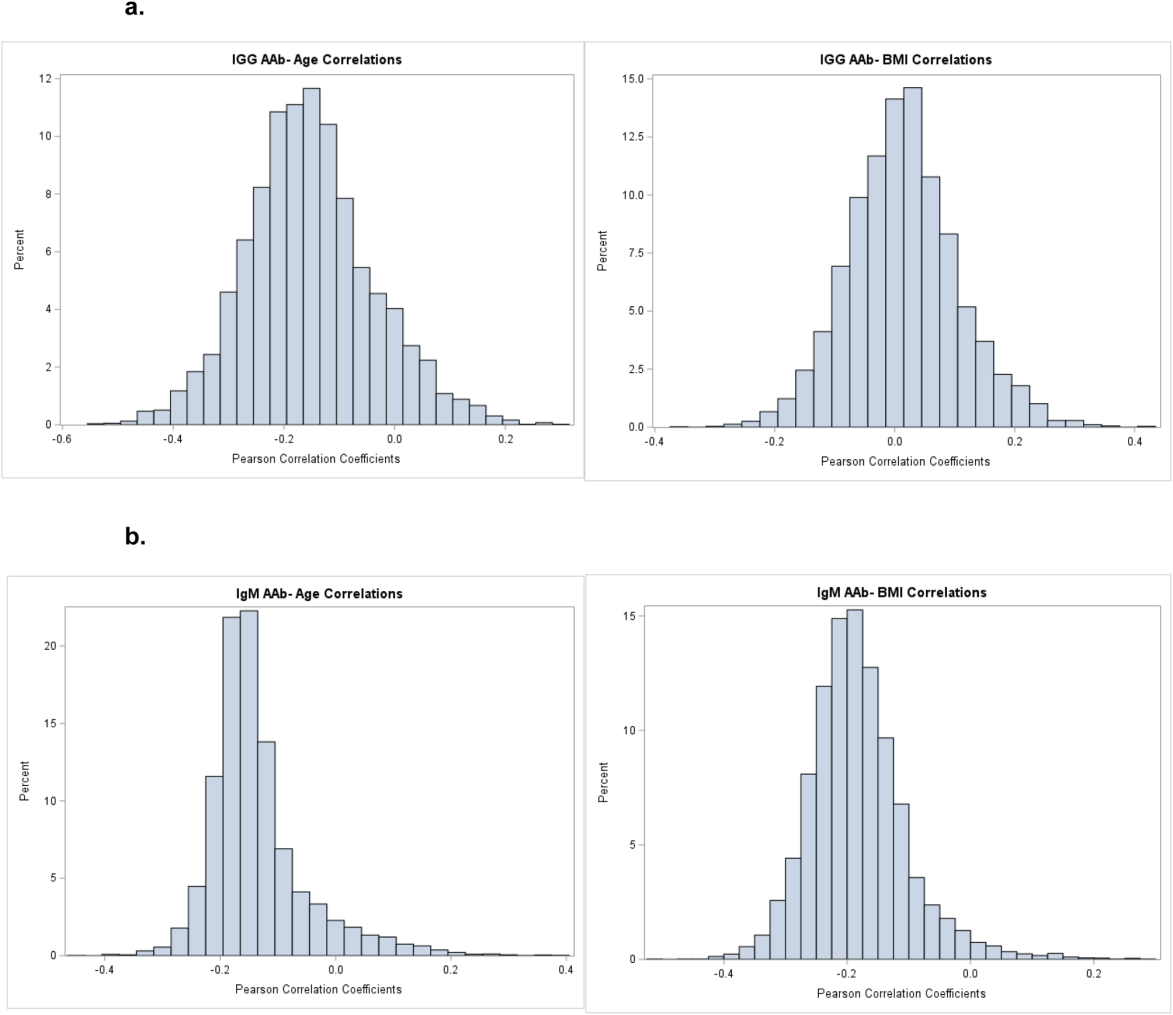
Distribution of Pearson correlation coefficients with age and BMI, restricted to autoantibodies with CV < 20% and ICC ≥ 0.6 (**a)** IgG correlation with age (left) and BMI (right). (**b)** IgM correlation with age (left) and BMI (right). Histograms of Pearson correlation coefficients for IgG (**a**) and IgM (**b**) AAbs with age (left panels) and BMI (right panels). Log2-transformed raw AAb concentrations correlations with age and BMI were between − 0.4 to 0.2 for nearly all IgG and IgM AAbs.

We assessed the correlation of AAb measurements with age and BMI. Most of the IgG and IgM AAbs were negatively correlated with age, and most IgM AAbs were negatively correlated with BMI. IgG AAbs were evenly spread between negative and positive correlations with BMI. However, none of the correlations of IgG or IgM AAbs with age or BMI were strong (correlation coefficients < │0.45│, Fig. 3).

**Figure 4:**
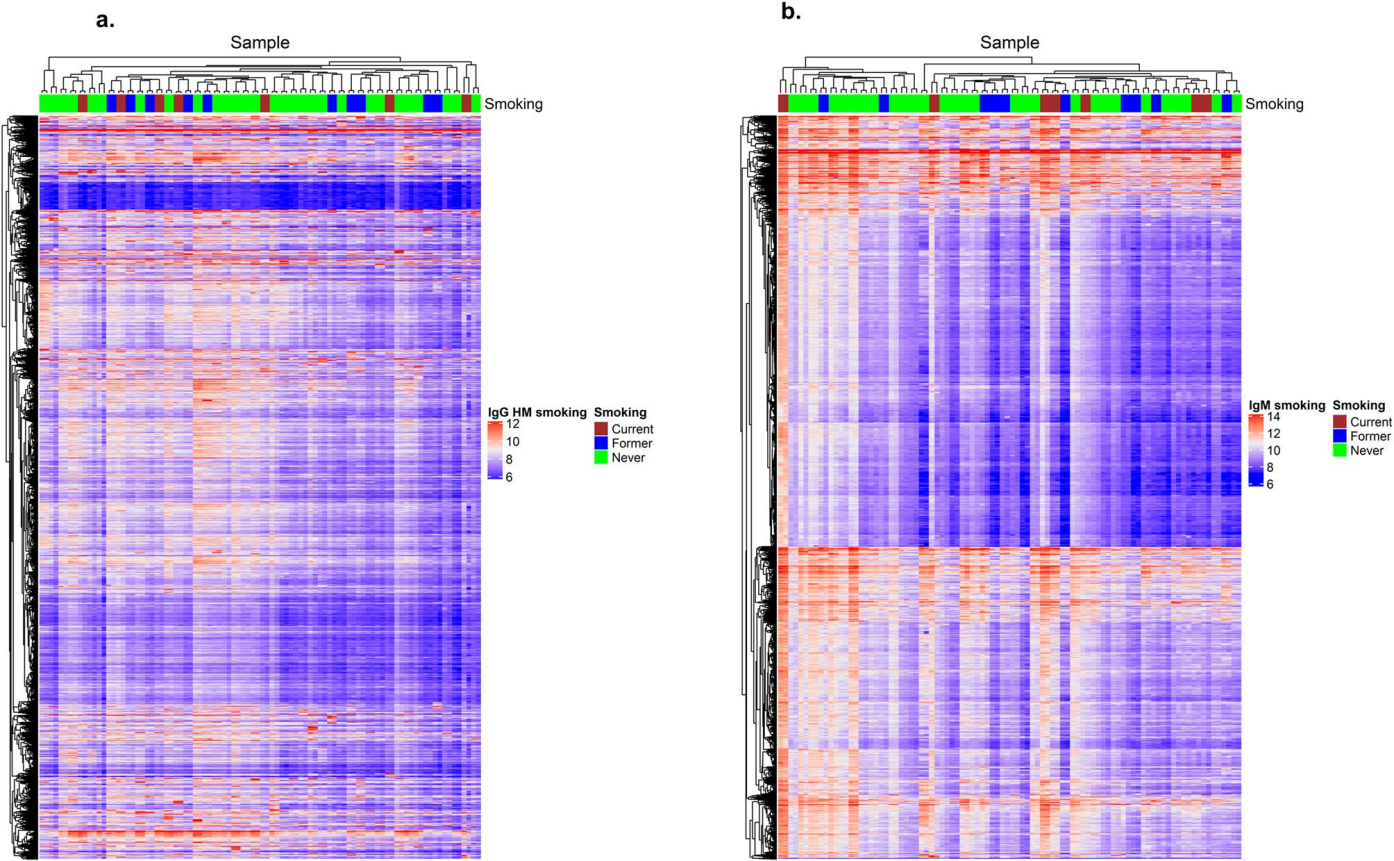
IgG (left) and IgM (right), by smoking status (never, former, current). IgG (**a**) and IgM (**b**) autoantibody profiling using HuProt arrays of repeat samples collected one year apart from *n* = 46 heathy subjects (*n* = 92 samples total). Raw log2 transformed data was used to cluster AAbs and samples using pearson distance higherarchical clustering for 18,303 IgG and 7,242 IgM AAbs with CV < 20%. Top bar annotation colors correspond to smoking status at blood donation (*n* = 7 current, 10 former, 29 never smokers). (Two samples from each subject clustered together for all 46 subjects, but did not cluster by smoking status).

We assessed whether samples would cluster according to smoking status using heatmaps of AAbs with CV < 20% and ICC ≥ 0.6. Smoking status (never, former, current) was not associated with AAb profiles (Fig. 4).

AAb-specific reproducibility estimates and average intensities for IgG and IgM are available in Appendices (see Supplementary IgG and IgM Files, Sheet 1). Those with CV < 20% and high ICC (> 0.8) both before and after quantile normalization are also shown along with information about their functional role (see Supplementary IGG File, Sheet 2). These AAbs showed a range of functions; for example, IgG AAbs were involved in signal transduction, energy production, cellular mobility, and gene expression regulation. One hnRNP (hnRNPD) and two histone (H2BC and H3A) IgGs were in the top 500 highest AAbs (as measured by mean intensity after log2 transformation), while most other hnRNPs, histones, GPI, SSAs, Sm, and other commonly expressed autoimmune-disease associated autoantibodies did not have high mean intensities. IgG AAbs that were commonly observed in previous studies of healthy subjects are shown along with our estimates in sheet 3 (IgM AAbs have not yet been studied in healthy individuals). We examined the temporal reproducibility of AAbs frequently observed in healthy individuals in other studies. 38 of the 66 IgG AAbs that were observed in at least 60% of participants in one study 4 had overlapping autoantigen probes on HuProt, and all but three had ICC > 0.6. We measured IgGs for eight of 12 other AAbs frequently observed in another study 19, and each of them had at least one probe with ICC > 0.6.

## Discussion

Most AAbs in serum samples stored at −80 °C for over 30 years could be measured with good assay precision using the HuProt array. We also found that samples collected one year apart from the same woman showed more similar AAb profiles than samples collected from different women, indicating that there is AAb repertoire stability in healthy individuals. When considered individually, a majority (> 70%) of IgG AAbs and a third of IgM AAbs had low laboratory variability (CV < 20%) and ICCs consistent with adequate to excellent temporal reproducibility among healthy women over a one-year period. Finally, clustering of samples from the same woman was better achieved, and proportion of AAbs with good temporal reliability higher, in analyses of the raw (log2-transformed) data than the quantile-normalized data.

Autoimmune and chronic diseases have been associated with specific AAb profiles^8,14,15^. As AAb profiles specific to autoimmune and other chronic diseases are identified and then refined to select the most informative panels associated with disease, ICCs should be considered as AAbs included in such panels should be stable in healthy individuals.

Comparing results from studies using different protein arrays is difficult because of the lability and 3-dimensional nature of proteins, differences in the methodology used to create the array, and in AAb nomenclature. However, our observations that many autoantibodies are detectable in healthy individuals and that an individual’s AAb profile is highly reproducible over time is consistent with the few other studies conducted to date. Nagele et al.^3^ examined about half as many AAbs (9486 IgG) using Invitrogen microarrays among 166 individuals, 57 without chronic disease, and observed the presence of hundreds to thousands of IgG AAbs, even among healthy individuals. Neiman et al.^16^ examined 335 protein fragments (204 proteins) selected for their known role in autoimmune disorders in 193 healthy individuals’ blood samples taken 4 times over a single year period. An AAb fingerprint was created for each sample, which was consistent over repeat timepoints. Four individual’s serum samples were further profiled using an array containing 23,000 protein fragments. For each of the 49 AAbs in these two studies considered to be frequently observed^3,16^ that were also measured in our study, all but three had at least one probe with high temporal reproducibility (see Supplementary IgG File, Sheet 3). Further, consistent with a previous study that examined temporal reproducibility of candidate AAbs individuals and those with cardiovascular disease^17^, we observed high ICCs for at least one probe for each of the seven AAbs common to our array that had reported high reproducibility (bottom of Supplementary IgG File, Excel Sheet 3).

IgG AAb differences among cancer cases vs. controls have been assessed using arrays, including HuProt^18^. Specifically, AAb biomarkers have been identified for lung, breast, esophogeal, gastric, ovarian, colorectal, and hepatic cell cancers^19–36^. The laboratory variability and temporal reproducibility estimates that we observed for these cancer markers are shown in the Supplementary IgG File (Sheet 4). Of the autoantigen probes for 34 HuProt-based markers from seven studies, 32 markers in our study had at least one autoantigen probe that showed high temporal reproducibility (all except for CLDN ICC: 0.40, PDGFRA ICC: 0.33, and one of five GNAS autoantigen probes ICC: 0.41). In 11 other studies of AAbs and cancer (not using HuProt), 50 AAbs were associated with one or more cancers. Of those, 45 were included in the array used in the present study, met laboratory quality control criteria, and were detected in the NYUWHS samples. Each of the 45, except four (TSC1 ICC: 0.47, two of four autoantigen probes for BDNF ICCs: 0.39, 0.45, one of five for FGFR2 ICC: 0.46, and one of two for PSRC1 ICC: 0.58) had ICCs > 0.6. Four of the markers identified from previous AAb studies that had low ICCs in our study, also had autoantigens for the same target with ICCs > 0.6. A strength of having more than one probe per marker on the array is that many markers are covered with redundancy, which may be important to consider in studies selecting optimal probe sets in future etiologic and early detection studies.

Normalization is commonly used in proteomic analyses, as array variability (also referred to as batch variability) is of concern^37,38^. Therefore, we conducted an analysis of the quantile-normalized data, in addition to the analysis of the raw (log2-transformed) data. Though it was not our expectation, clustering of samples from the same woman was not as good using quantile-normalized data as raw data, suggesting that batch variability is not as much of a concern, at least for the HuProt assay, as previously thought. While normalization should be assessed as part of a standard analysis to address concerns of batch variability, it may obscure some true biological differences.

Our study has several strengths. To our knowledge, it is the largest study to assess the occurrence and temporal reproducibility of AAbs among individuals who were free of cancer and cardiovascular disease not only at the two blood draws, but also in the following 35 years. For many proteins, the HuProt array includes several antigens, providing redundancy and capturing some AAbs to fragmented or variant antigens. This is also the first study that assessed reproducibility of an array of IgM AAbs. The inclusion of eight replicates of a pooled sample interspersed in a blinded fashion within the paired samples allowed us to accurately assess assay precision. CVs were good for most AAbs and suggest that batch effects were not a major concern, particularly for IgG assays. Further, despite not controlling for non-specific binding, we still observed high intra-subject stability in samples collected over time for most AAbs, indicating that noise from sticky binding did not obscure autoantibody profiles.

A limitation of our study is that the sample size was adequate to estimate temporal reproducibility but was insufficient to reliably determine the impact of subject characteristics, such as demographic and lifestyle variables, on the AAb profile. It is not clear why a high proportion of IgM AAbs had high laboratory variability (CV > 20%) despite being assayed simultaneously in the same array. It is possible that both biological and technical factors contributed, including lower specificity binding of most IgM AAbs, the larger size of IgM vs. IgG AAbs, and the relationship of these factors to array characteristics (e.g. antigen spacing)^39^. Further, all of the antigens spotted on the array are proteins and protein fragments. Because these proteins are expressed in yeast, some post-translational modifications (e.g., glycosylation and phosphorylation) will be present and printed on the array. However, the arrays we used would not have captured autoantibodies directed at non-protein targets, such as DNA, oxidized lipids and other frequent non-protein targets of autoantibodies as well as an extensive array of citrullinated, acetylated, and other post-translationally modified proteins. The number and types of AAb targets are vast and may be included on arrays specific to the target type in the future^40^.

In conclusion, our results show that many AAbs are present and detectable in healthy women and that their levels are stable over a one-year period. AAbs in the blood reflect systemic autoimmunity and can be measured rapidly in large numbers using arrays. Our observations, along with the established role of AAbs in health and disease, suggest that they may be good candidate biomarkers of risk and early detection of cancer and other chronic diseases.

## Supplementary Information

Supplementary Information 1.

Supplementary Information 2.
